# Supplementary material for: Luminex MFI—Efforts from a Qualitative to a Quantitative Analysis
Source: Biology (Basel). 2025 Jun 12;14(6):686. doi: 10.3390/biology14060686 (PMC12189511; doi:10.3390/biology14060686)
Supplement: Supplementary file 1 [file biology-14-00686-s001.zip › biology-3599335-supplementary.pdf]

biology

MDPI

Supplementary Materials:

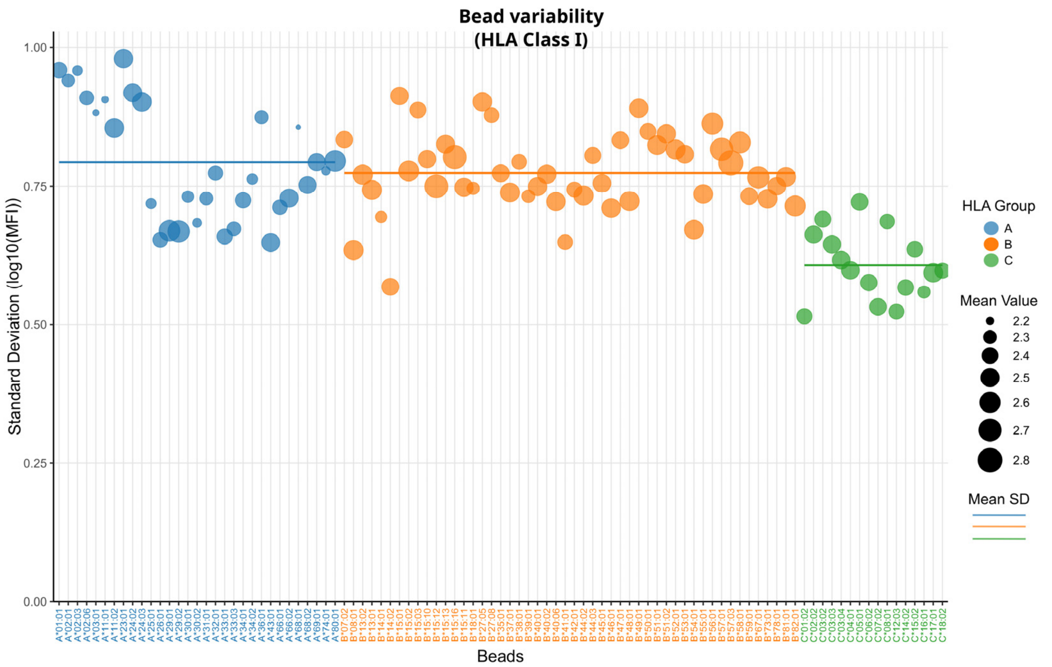

Figure S1*:* HLA class I bead variability. Plot analogue to Figure 2 without the 1,500 MFI filter. The Y-axis shows the standard deviation of log10-transformed MFI values of each bead. Each dot represents a bead, with its size proportional to the mean MFI value of all measurements. Dots are colored according to HLA locus, horizontal lines represent the mean SD for each locus, respectively.

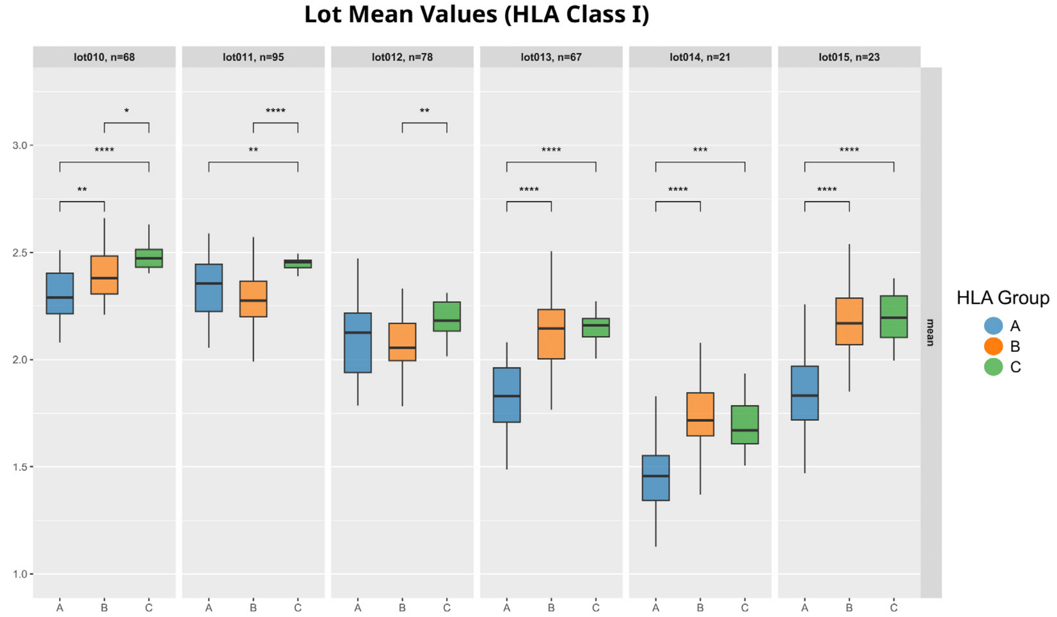

Figure S2: Lot-to-lot variability of mean MFI values from HLA class I beads. Each box represents SD in log10 of one HLA class I locus, and the loci are grouped by LABScreenSingle Antigen lot. Significant differences between the HLA loci were identified using Wilcoxon rank-sum test without Bonferroni correction (significance codes: **** p < 0.0001, *** p < 0.001, ** p < 0.01, * p < 0.05).

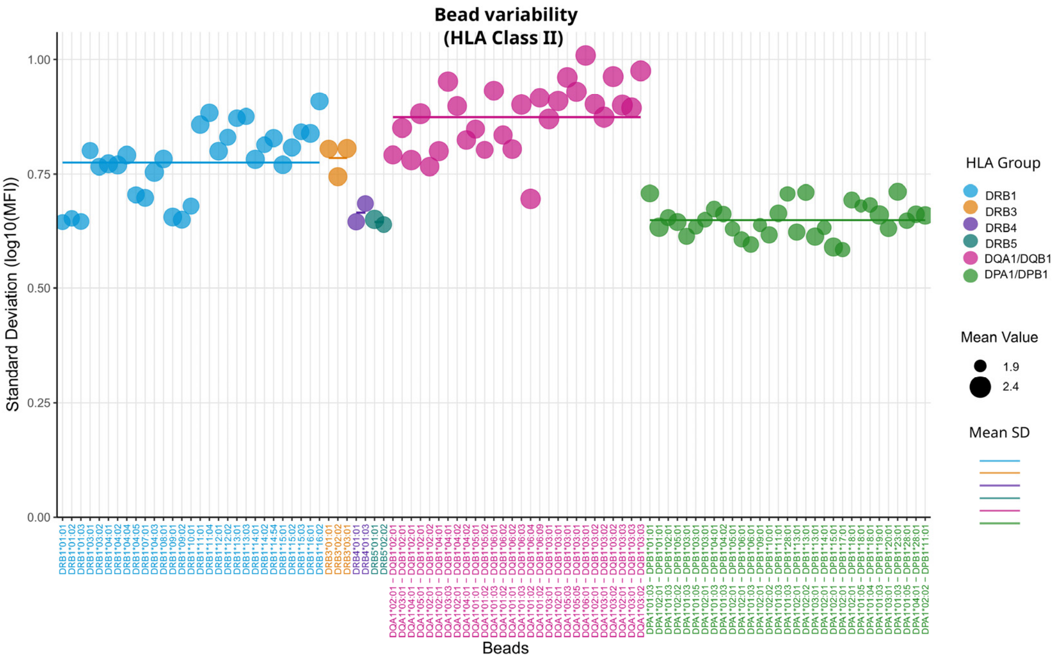

Figure S3 Bead variability of HLA class II without 1,500 MFI filter: The Y-axis shows the standard deviation of log10 transformed MFI values of each bead. Each dot represents a bead, with its size proportional to the mean MFI value of all measurements. Dots are colored according to HLA locus, horizontal lines represent the mean SD for each locus, respectively.

Table S1: Cohort characteristics

Patient characteristics	Recipients (n=61)
Age (y), mean ± SD	36.2 ± 18.9
Sex female n (%)<br>Sex male n (%)	27 (44.3)<br>34 (55.7)
Transplantation period	1990 - 2025
**Transplanted organs/ Therapy (n)**	
Stem cell	2
Bortezomib therapy	1
liver	18
living liver	1
kidney postmortal	16
living kidney	21
kidney-pancreas	2
Multiple transplants	14

Table S2: Bead coating for HLA class I beads. Colors indicate the HLA locus according to Figure 2.

A*01:01					
A*02:01	A*02:03	A*02:06	A*03:01	A*11:01	A*11:02
A*23:01	A*24:02	A*24:03	A*25:01	A*26:01	A*29:01
A*29:02	A*30:01	A*30:02	A*31:01	A*32:01	A*33:01
A*33:03	A*34:01	A*34:02	A*36:01	A*43:01	A*66:01
A*66:02	A*68:01	A*68:02	A*69:01	A*74:01	A*80:01
B*07:02	B*08:01	B*13:01	B*13:02	B*14:01	B*14:02
B*15:01	B*15:02	B*15:03	B*15:10	B*15:11	B*15:12
B*15:13	B*15:16	B*18:01	B*27:05	B*27:08	B*35:01
B*37:01	B*38:01	B*39:01	B*40:01	B*40:02	B*40:06
B*41:01	B*42:01	B*44:02	B*44:03	B*45:01	B*46:01
B*47:01	B*48:01	B*49:01	B*50:01	B*51:01	B*51:02
B*52:01	B*53:01	B*54:01	B*55:01	B*56:01	B*57:01
B*57:03	B*58:01	B*59:01	B*67:01	B*73:01	B*78:01
B*81:01	B*82:01	C*01:02	C*02:02	C*03:02	C*03:03
C*03:04	C*04:01	C*05:01	C*06:02	C*07:02	C*08:01
C*12:03	C*14:02	C*15:02	C*16:01	C*17:01	C*18:02

Table S3: Bead coating for HLA class II beads. Colors indicate the HLA locus according to Figure 5

DRB1*01:01	DRB1*01:02	DRB1*01:03
DRB1*03:01	DRB1*03:02	DRB1*04:01
DRB1*04:02	DRB1*04:04	DRB1*04:05
DRB1*07:01	DRB1*04:03	DRB1*08:01
DRB1*09:01	DRB1*09:02	DRB1*10:01
DRB1*11:01	DRB1*11:04	DRB1*12:01
DRB1*12:02	DRB1*13:01	DRB1*13:03
DRB1*14:01	DRB1*14:02	DRB1*14:54
DRB1*15:01	DRB1*15:02	DRB1*15:03
DRB1*16:01	DRB1*16:02	DRB3*01:01
DRB3*02:02	DRB3*03:01	DRB4*01:01
DRB4*01:03	DRB5*01:01	DRB5*02:02
DQA1*02:01+DQB1*02:01	DQA1*03:01+DQB1*02:01	DQA1*04:01+DQB1*02:01
DQA1*05:01+DQB1*02:01	DQA1*02:01+DQB1*02:02	DQA1*02:01+DQB1*04:01
DQA1*03:03+DQB1*04:01	DQA1*02:01+DQB1*04:02	DQA1*04:01+DQB1*04:02
DQA1*01:01+DQB1*05:01	DQA1*01:02+DQB1*05:02	DQA1*01:03+DQB1*06:01
DQA1*01:02+DQB1*06:02	DQA1*01:01+DQB1*06:02	DQA1*01:03+DQB1*06:03
DQA1*01:02+DQB1*06:04	DQA1*01:02+DQB1*06:09	DQA1*03:01+DQB1*03:01
DQA1*02:01+DQB1*03:01	DQA1*05:03+DQB1*03:01	DQA1*05:05+DQB1*03:01
DQA1*06:01+DQB1*03:01	DQA1*02:01+DQB1*03:02	DQA1*03:01+DQB1*03:02
DQA1*03:02+DQB1*03:02	DQA1*02:01+DQB1*03:03	DQA1*03:01+DQB1*03:03
DQA1*03:02+DQB1*03:03	DQA1*05:05+DQB1*03:19	DQA1*03:03+DQB1*03:02
DPA1*01:03+DPB1*02:01	DPA1*02:02+DPB1*05:01	DPA1*01:03+DPB1*03:01
DPA1*01:05+DPB1*03:01	DPA1*02:01+DPB1*03:01	DPA1*01:03+DPB1*04:01
DPA1*01:03+DPB1*04:02	DPA1*02:01+DPB1*05:01	DPA1*02:01+DPB1*06:01
DPA1*01:03+DPB1*06:01	DPA1*02:01+DPB1*09:01	DPA1*02:02+DPB1*10:01
DPA1*01:03+DPB1*11:01	DPA1*01:03+DPB1*28:01	DPA1*02:01+DPB1*13:01
DPA1*02:02+DPB1*13:01	DPA1*03:01+DPB1*13:01	DPA1*02:01+DPB1*14:01
DPA1*02:01+DPB1*15:01	DPA1*02:01+DPB1*17:01	DPA1*02:01+DPB1*18:01
DPA1*01:05+DPB1*18:01	DPA1*01:04+DPB1*18:01	DPA1*01:03+DPB1*19:01
DPA1*03:01+DPB1*20:01	DPA1*01:03+DPB1*23:01	DPA1*01:05+DPB1*28:01
DPA1*04:01+DPB1*28:01	DPA1*02:02+DPB1*11:01	DPA1*01:03+DPB1*01:01
DPA1*02:01+DPB1*01:01		

Table S4. Results from Wilcoxon rank sum tests that have significant SD differences ($p < 0.05$). Table shows p value with Bonferroni correction, effect size (r), Confidence Intervals (CI). In the Class II group, comparisons without significant differences were excluded.

Dataset	Comparison	P (adj.)	r	CI (95%)
Class I – Filter (Figure 2)	A-C	7.59e-7	0.681	[0.038, 0.079]
	A-B	1.00e-03	0.395	[0.013, 0.046]
	B-C	3.00e-03	0.402	[0.012, 0.045]
Class II – No Filter (Figure S1)	A-C	4.56e-08	0.727	[0.118, 0.246]
	A-B	1	0.040	[–0.037, 0.060]
	B-C	7.5e-08	0.687	[0.130, 0.208]
Class II – Filter (Figure 5)	DRB1-DPA1/DPB1	5.00e-03	0.452	[–0.052, -0.015]
Class II – No Filter (Figure S3)	DPA1/DPB1-DQA1/DQB1	6.51e-15	0.849	[–0.261, -0.194]
	DPA1/DPB1 -DRB1	1.03e-07	0.687	[–0.168, -0.101]
	DQA1/DQB1 - DRB1	2.02e-04	0.552	[0.054, 0.137]
	DPA1/DPB1 - DRB3	5.00e-03	0.484	[-0.183, -0.094]

Table S5: Variability of HLA class I and HLA class II beads without 1,500 MFI filter. Mean and SD are calculated in log10. Fold change is the retransformed SD in linear space (10^{SD}).

HLA-Locus	Mean	SD	Fold change
A	2.37	0.79	6.17
B	2.50	0.77	5.89
C	2.41	0.60	3.98
DPA1/DPB1	2.26	0.65	4.47
DQA1/DQB1	2.63	0.88	7.59
DRB1	2.34	0.78	6.02
DRB3	2.43	0.79	6.17
DRB4	2.26	0.67	4.67
DRB5	2.36	0.65	4.47

Disclaimer/Publisher's Note: The statements, opinions and data contained in all publications are solely those of the individual author(s) and contributor(s) and not of MDPI and/or the editor(s). MDPI and/or the editor(s) disclaim responsibility for any injury to people or property resulting from any ideas, methods, instructions or products referred to in the content.
